# Toothbrushing: to the best of one’s abilities is possibly not good enough

**DOI:** 10.1186/s12903-018-0633-0

**Published:** 2018-10-19

**Authors:** Renate Deinzer, Stefanie Ebel, Helen Blättermann, Ulrike Weik, Jutta Margraf-Stiksrud

**Affiliations:** 1 Department of Medicine, Justus-Liebig-University Giessen, Klinikstr. 29, D-35392 Giessen, Germany; 2 Department of Psychology, Philipps University of Marburg, Gutenbergstr. 18, D-35032 Marburg, Germany

**Keywords:** Oral hygiene, Community dentistry, Dental education, Dental hygiene, Preventive dentistry, Behavioral science, Toothbrushing

## Abstract

**Background:**

Weaknesses in toothbrushing performance can be seen when young adults are instructed to perform habitual toothbrushing. Nothing is known about toothbrushing behavior when instructed to perform to the best abilities. The present study analyzes such behavior and compares it to habitual behavior.

**Methods:**

A random sample of *N* = 98 young adults born in 1995 was examined in 2014/2015.They were asked to perform oral hygiene to the best of their abilities in front of a camera. Videos were analyzed regarding details of brushing behavior. A quality index was developed which describes the extent of the neglect of brushing on palatinal and vestibular surfaces. Data were compared to those of an earlier study of young adults (born in 1992, examined in 2011, *N* = 101) who were asked to perform oral hygiene as they habitually do.

**Results:**

The 1995 cohort (best abilities) brushed their teeth significantly longer than the 1992 cohort (habitual brushing). This was due to significant longer brushing at vestibular and occlusal surfaces. Neglect of palatinal surfaces was similar in both cohorts. Groups did not differ regarding brushing movements. 40% of the brushing time on lateral surfaces was spent with scrubbing movements despite opposing advice in common oral hygiene instructions.

**Conclusions:**

Toothbrushing to the best of one’s abilities might still not be good enough. Young adults apparently lack a reasonable concept of what is meant by high quality toothbrushing. More efforts should thus be undertaken to explain them (and adults) this concept.

**Electronic supplementary material:**

The online version of this article (10.1186/s12903-018-0633-0) contains supplementary material, which is available to authorized users.

## Background

There is hardly any other health behavior in western communities that is performed by such a great portion of the population on a regular basis as oral hygiene behavior. This can be considered a major success of joint ventures by dental health professionals, health politicians and educational staff, who succeeded in establishing oral hygiene as a daily routine in the vast majority of the population [1, 2]. Still, oral diseases, especially gingivitis and periodontitis, are very common in adults. Nearly everyone suffers from gingivitis and approximately 50% show at least some periodontal breakdown [2–6]. It thus appears that even though oral hygiene is performed on a regular basis, its quality tends to be low or at least insufficient with respect to oral health maintenance. A series of recent studies has shown that adults hardly ever manage to clean more than 30–40% of their gingival margins by means of tooth brushing and interproximal hygiene [7–12]. Thus, the question arises of why oral hygiene behavior is so inefficient.

In order to answer this question, a recent study analyzed the oral hygiene behavior of young adults who had just come of age [13]. On reaching this age, years of prophylactic programs aiming to bring them into a position of maintaining oral health during adulthood had concluded. These young adults were asked to demonstrate their habitual oral hygiene behavior while being filmed. Analyses showed that they brushed their teeth for more than 2 min, but distributed brushing time unevenly among sextants and surfaces and neglected palatinal and lingual surfaces. Furthermore, even though the prophylactic programs teach children and adolescents to apply circular and/or vertical rather than horizontal brushing movements at lateral surfaces, horizontal movements were evident in most of them for a considerable period of time. Finally, only 50% showed some interproximal hygiene behavior. In addition, this was considered inadequate in most cases [13].

While this study gave important insights into habitual oral hygiene behavior of young adults, nothing was learned about their behavior when they perform to the best of their abilities. From additional analyses it is already known that even then they do not manage to achieve oral cleanliness at most of their gingival margins [9]. However, nothing is known about what characterizes their oral hygiene behavior under such circumstances and whether and in what respect it differs from their habitual behavior.

The present study thus aimed to investigate young adults’ oral hygiene behavior when they were asked to perform oral hygiene to the best of their abilities and to analyze behavioral differences from the former cohort of young adults [13] who had been asked to perform their habitual oral hygiene behavior.

## Methods

The aim of the present study was to analyze oral hygiene behavior of young adults when they were asked to perform to the best of their abilities and to compare this behavior to behavior assessed in an earlier study [13] when participants were asked to perform as usual.

### Participants and general design

Methods relevant for the comparison of the former cohort [13] and the current one were essentially the same and a detailed description will only be given for the current cohort. The study protocol of the former study has been described in detail elsewhere [13]. In order to enhance the comparability to the previous study [13], this study assessed oral hygiene behavior in young adults living in the same town (approx. 80,000 inhabitants) in central Germany. All inhabitants born in 1995 were invited. They had come of age a few months prior to the beginning of recruitment in August 2014. The assessments took place in dental examination rooms of the Institute of Medical Psychology, Justus-Liebig-University Giessen, Germany, from August 2014 to July 2015.

*N* = 98 young adults (43 males and 55 females) participated in the present study. Details of recruitment are shown in the Additional file 1.

All participants fulfilled the inclusion criteria: 1) born in 1995; 2) being a resident of Giessen; 3) no training in any dental profession; 4) providing informed written consent, and also the exclusion criteria: 1) fixed orthodontic appliances; 2) cognitive or physical impairment that affects toothbrushing; 3) habitual use of a powered toothbrush; 4) removable dentures.

Participants were placed in front of a wash basin and a tablet computer. The tablet computer had an integrated front camera and it served both as a mirror for the participants and as a camera. All participants were provided with a standardized toothbrush and toothpaste. In contrast to the earlier study where participants were only offered 0.5 m of dental floss, the present participants were provided with a selection of different means for proximal hygiene (waxed and unwaxed dental floss, superfloss, interdental brushes). Participants were asked to clean their teeth to the best of their abilities and were left alone while performing oral hygiene. In the present study, some clinical data were also assessed prior to and immediately after oral hygiene. Most importantly, the marginal plaque index (MPI) [11] was assessed immediately after oral hygiene by a calibrated examiner (for details see Ebel et al. submitted). No corresponding data are available from the previous study [13]. Clinical data are discussed in detail elsewhere [14]. Furthermore, some questionnaire data were assessed regarding psychological parameters which will not be discussed in the current analyses. The highest degree of education of the participants’ parents was assessed as a measure of socioeconomic status. Two categories were formed for analyses: university entrance diploma or not.

### Observed oral hygiene behavior

The videos were analyzed by two independent calibrated examiners (HB und SE) using the software Mangold Interact 14 (Mangold International GmbH, Arnstorf, Germany). Brushing hand was coded and the examiners watched the video multiple times (also in slow motion) in order to code further behavioral categories. Calibration was provided by five videos of individuals not involved in the present study.

In adulthood, caries manifests primarily at lateral and proximal surfaces [15–17]. The risk of developing gum disease and periodontitis increases and exceeds that of caries [2, 5]. Therefore, special emphasis was given to brushing behavior at lateral surfaces. Analyses regarding brushing movements and precise localization of brushing (sextants) were confined to them. To ensure that these analyses were not contaminated by occlusal brushing, lateral brushing was only assumed if both raters agreed that it was not occlusal. Thus, both examiners coded tooth contact time (time while toothbrush touches the teeth, without rinsing, spitting, tongue cleaning or breaks) and surfaces (vestibular, palatinal and occlusal) for all participants. SE carried out all further ratings and confined these to the palatinal and vestibular surfaces. These were: brushing movements (horizontal i.e. scrubbing, vertical, circular, modified Bass technique) and sextants (sextant 1 to 6, and, at vestibular surfaces, also concurrent brushing of antagonistic sextants, i.e. 1 and 6, 2 and 5, 3 and 4, respectively). Concurrent brushing of antagonistic sextants was coded when participants closed the mandibles while brushing. For further analyses, the brushing time of two sextants brushed concurrently was distributed in equal parts to both sextants.

To assess whether codings of sextants and movements remained reliable over time, double codings of a random sample of films were performed by HB. SE did not know which films were double coded and HB did not know SE’s codings. Intraclass correlations of double codings of the various observational categories were all above 0.801.

In the former study [13], all behavioral parameters were assessed by two independent examiners. Their intraclass correlations regarding the different parameters exceeded ICC = 0.865. For the present analyses, we computed the mean values of these double codings, since aggregating double codings further increases their reliability.

It was also intended to assess interdental cleaning (i.e. any application of devices for interdental cleaning in interdental spaces). However, only 15 participants performed interdental cleaning. Furthermore, most of these applied them only in some interdental spaces. We therefore refrained from any further analysis of this behavior.

A main result of the former study was that participants neglected surfaces and sextants while brushing. In the present analysis, a scoring system was thus developed allowing further description and analysis of the quality of toothbrushing at palatinal and vestibular surfaces (Quality index of toothbrushing regarding brushing time in sextants: QIT-S; for details see Table 1).

**Table 1:** The Quality index of toothbrushing regarding brushing time in sextants (QIT-S^a^)

QIT-S-0	0 sextants brushed by more than 1 s
QIT-S-1	1 sextants brushed by more than 1 s
QIT-S-2	2 sextants brushed by more than 1 s
QIT-S-3	3 sextants brushed by more than 1 s
QIT-S-4	4 sextants brushed by more than 1 s
QIT-S-5	5 sextants brushed by more than 1 s
QIT-S-6	all sextants brushed by more than 1 s
QIT-S-7	all sextants brushed by more than 3.5 s
QIT-S-8	all sextants brushed by more than 5 s
QIT-S-9	all sextants brushed by more than 7.5

^a^The QIT-S index represents a rank-scaled measure allowing for the differential analysis of brushing time distribution at palatinal and vestibular sites, respectively. Brushing time of less than 1 s within a sextant is considered neglect of this sextant. QIT-S-0 ─ QIT-S-6 describe the expansion of this neglect. The highest score (QIT-S-9) is deduced from a recommended total brushing time of 2 min (e.g. 19 [19]; 18 [18]; 20 [20]) and an estimation of 30 s brushing time for occlusal surfaces as occlusal brushing is easier and can be done with greater movements than palatinal or vestibular brushing. Thereby, 45 s remain for the palatinal and vestibular surfaces, respectively. An even distribution of this time across sextants results in 7.5 s per sextant. As 3.5 represents roughly half of 7.5 this is taken as further step (QIT-S-7) and as 5 s per sextant might already be considered fair this was taken as another step (QIT-S-8)

### Statistics

The intended significance level was α = 5%. For parametric data, comparisons within a cohort were performed by paired t-tests and group comparisons by t-tests for independent measures. The latter are reported together with Cohen’s d. If Levene’s test for comparison of variances reached the significance level, the t-statistic for unequal variances was chosen. For non-parametric data, results of exact rank tests or exact Chi^2^ tests are reported. All analyses were run with IBM SPSS Statistics Version 24.

## Results

For ease of reading, the current cohort will be the 1995 cohort and the former cohort [13] will be referred to as the 1992 cohort. Two participants of the 1995 cohort had to be excluded from further analyses: one due to restricted visibility during brushing, another because of her extremely extended brushing time (573.4 s, 4 standard deviations above the mean) which also led to distortions of the reliability estimates of observational measures.

In the 1995 cohort, percentage of marginal sections showing persistent plaque (MPI; 11) immediately after oral hygiene was 69.4% ±12.3 regarding the whole mouth, and 60.9% ± 15.3 and 78.0% ± 12.2 regarding vestibular and palatinal sites, respectively. No such data is available for the 1992 cohort.

Table 2 shows the characteristics of the two cohorts with respect to sex, parents’ education and brushing hand. No significant differences between the cohorts were found.

**Table 2:** Characteristics of the two cohorts

	1995 cohort	1992 cohort	p (exact test)
sex	male/female	male/female	0.491
	42/54	43/58	
at least one parent has university entrance diploma (UED)	no UED/UED	no UED/UED	0.085
	36/60	51/50	
brushing hand in video	right/left/both	right/left/both	0.886
	85/7/4	85/10/6	

Table 3 shows the brushing behavior of the two cohorts. Both cohorts spent most of the brushing time on vestibular and occlusal surfaces. Significantly less brushing time was spent on palatinal than on vestibular surfaces in both cohorts (all *p* < .001). Groups did not differ with respect to brushing movements on lateral surfaces. Circular and horizontal brushing predominated, while vertical brushing was rarely shown for longer periods of time. Only 1 person of the 1995 cohort applied the modified Bass technique.

**Table 3:** Brushing behavior within the two cohorts

	1995 cohort (*N* = 96) Mean (SD)	1992 cohort (*N* = 101) Mean (SD)	t (195)	d	p
Tooth contact time (s)					
overall	206.7 (84.0)	155.3 (70.8)	4.66	.664	<.001
vestibular	91.3 (40.7)	70.9 (31.2)	3.94	.565	<.001
palatinal	30.6 (30.9)	26.0 (27.9)	1.10	.157	.272
occlusal	84.8 (46.0)	58.4 (34.2)	4.55	.654	<.001
Percentage of time lateral surfaces are brushed by					
horizontal (scrubbing) brushing movements	39.7 (28.8)	39.7 (30.3)	.002	<.001	.998
circular brushing movements	47.0 (27.3)	44.8 (29.5)	.548	.078	.584

**Fig. 1:**
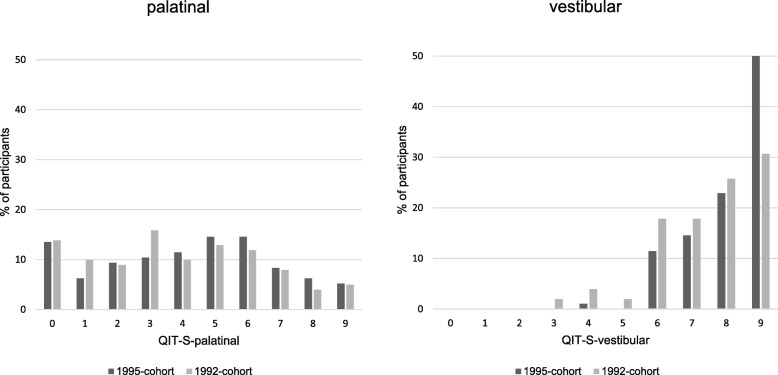
Histogram of QIT-S scores at palatinal and vestibular sites, respectively. The 1992 cohort had been asked to brush their teeth like they commonly do while the 1995 cohort had been asked to brush their teeth to the best of their abilities. A statistical significant cohort difference is found for vestibular sites, only. QIT-S palatinal is significantly lower than QIT-S vestibular in both cohorts

Figure 1 shows the QIT-S scores. QIT-S palatinal was significantly lower than QIT-S vestibular in both groups (exact *p* < .001). Groups did not differ with respect to QIT-S palatinal (exact *p* = .331) while the 1992 cohort showed lower QIT-S vestibular scores than the 1995 cohort (exact *p* = .002).

A further analysis was run in order to better understand the differences between the cohorts regarding vestibular brushing: The percentage by which these groups distributed their vestibular brushing time to right (sextants 1 and 6), left (sextants 2 and 5) and anterior (sextants 3 and 4) surfaces was compared. Cohorts did not differ with respect to brushing time at left surfaces, but the 1995 cohort showed prolonged brushing of anterior and right surfaces (see Table 4).

**Table 4:** Brushing time across vestibular surfaces

	1995 cohort (*N* = 96) Mean (SD)	1992 cohort (*N* = 101) Mean (SD)	t (195)	d	p
Brushing time (seconds)					
anterior	39.3 (21.3)	24.7 (15.6)	5.5	.788	<.001
right	26.6 (15.4)	19.7 (10.2)	3.7	.532	<.001
left	25.2 (13.9)	26.5 (13.5)	─0.6	─.088	.536

## Discussion

One important aim of oral health education of children and adolescents is to enable them to employ proper oral hygiene until they come of age. However, recent studies have shown that the plaque removal capability of young adults still tends to be low [7–10]. A major aim of the present study was thus to explore their oral hygiene skills. Therefore, participants were observed while performing oral hygiene to the best of their abilities. In order to differentiate their behavior with respect to distribution of brushing time, a quality index (QIT-S) was developed.

The good news from these observations is that young adults, when asked to perform oral hygiene to the best of their abilities, spent an average of 3:20 min brushing. This is more than 60% above common recommendations (see for example [18–20]) and suggests that they were motivated to give their best.

The bad news is that they distributed this brushing time neither evenly nor efficiently: These young adults brushed occlusal surfaces nearly 3 times longer than palatinal surfaces, even though gum disease and even caries in adults [15–17] originate at lateral surfaces. Furthermore, 80% of the study sample skipped at least one sextant when brushing palatinal surfaces (QIT-S palatinal ≤5); only 5% brushed all palatinal sextants for more than 7.5 s (QIT-S palatinal = 9). Vestibular sextants, on the other hand, were hardly ever skipped (only by one person), and were brushed for more than 7.5 s (QIT-S vestibular = 9) by 50% of the study sample.

Regarding brushing techniques, oral hygiene education usually teaches children to brush lateral surfaces either by circular or by vertical but not by scrubbing movements (see for example [21–23]). Still, the present sample spent nearly 40% of the brushing time on lateral surfaces scrubbing.

Considering that the participants of this study performed oral hygiene to the best of their abilities, the question arises as to what characterizes this behavior “to the best of one’s abilities” as compared to one’s common behavior. A second aim of the present study was thus to compare its results to a former study which had analyzed young adults’ common oral hygiene behavior [13]. This comparison elicited three important results: First, even though the total brushing time of the “to the best of one’s abilities” group exceeded that of the “common oral hygiene” group by more than a minute, the time spent brushing palatinal surfaces remained the same. Secondly, maximizing one’s efforts apparently did not result in altering one’s brushing technique. The cohorts did not differ with respect to the applied brushing movements. The only improvement of quality in oral hygiene behavior was seen with respect to vestibular surfaces: Neglect of surfaces (QIT-S vestibular ≤5) decreased, whereas the portion of participants achieving the highest quality score (QIT-S vestibular = 9) increased (see Fig. 1). A closer inspection of the distribution of brushing time across vestibular surfaces indicated that maximizing one’s efforts resulted in a disproportionately high increase in brushing time of anterior surfaces: Interestingly, these surfaces were already brushed for the longest time when people were asked to show their “common” hygiene behavior.

Summarizing, these results indicate that young adults, when asked to brush their teeth to the best of their abilities, tend to increase efforts within regions they already brush for a disproportionately long time (i.e. occlusal sites, vestibular sites, and within vestibular sites, anterior teeth), but continue to neglect palatinal sites. Furthermore, a considerable portion of brushing time remains spent scrubbing, irrespective of opposing content of oral hygiene teaching.

Considering these results, one has to doubt that young adults have adopted a reasonable concept of what is meant by high quality oral hygiene behavior. Their concept appears to be confined to brushing time. Neither did they seem to be aware of the meaning of brushing systematics (in order not to forget any surfaces), nor did they alter brushing techniques. The first point is especially striking, since neglecting whole regions while brushing inevitably results in poor plaque removal. Regarding the second point, one should keep in mind that strong scientific evidence demonstrating the superiority of one brushing technique above another is lacking [7, 24]. Still, dental advice commonly discourages people from scrubbing. Thus, one would have expected that the percentage of time spent scrubbing decreases when people try to perform high quality brushing.

Some limitations of the present study should be considered. First of all, self-selection of the participants may have biased results. This, however, presumably resulted in an overestimation of the toothbrushing quality of the cohort, as one would expect mainly those who doubt their competence to reject participation in an oral hygiene study. Secondly, it remains unclear as to what degree study results can be generalized to other regions of the world. Instead, it would be worth exploring whether similar or differing results would be observed in other nations with differing oral hygiene education programs. The present research demonstrates how important it is to analyze oral hygiene behavior more closely in order to understand hygiene deficits. Thirdly, the comparison between the two cohorts is merely quasi-experimental, thus not allowing for firm causal conclusions. While groups are perfectly comparable regarding age at the time of examination and with respect to demographic characteristics, concerns might arise regarding the following factors: year in which the participant was born and the examination took place, oral hygiene devices, different examiners. However, there is only a three-year gap between the two studies, the hygiene devices were very similar, and all examiners were calibrated by the same method and very good intraclass correlations were achieved. Thus, the comparability of the cohorts appears to be good enough to justify at least some reasoning about the meaning of the instruction (to the best of one’s abilities vs. common behavior) for oral hygiene behavior. Finally, one might question whether the behavioral deficits observed here reflect deficits in oral hygiene motivation rather than in oral hygiene skills. However, participants performed oral hygiene in a dental setting, they were asked to perform to the best of their abilities, they knew that they would be given a clinical examination afterwards and they brushed their teeth for far longer than usually recommended. This all argues against the assumption that the study results reflect motivational deficits rather than skill deficits.

Still, future research is needed to overcome the limitations of this analysis. Most importantly, the effect of different oral hygiene instructions should be assessed within a randomized controlled trial (RCT) and plaque after oral hygiene should be assessed in that study and related to hygiene behavior. Future studies should also focus on other age groups.

## Conclusion

Concluding, the present study demonstrates that - at least in young German adults - the demand to improve one’s oral hygiene might be useless as long as it is not explained in detail what exactly has to be improved. The observed distribution of brushing time across regions indicates that young adults have a poor concept of what is important while brushing. They appear to prefer those sites which are visible (vestibular, mainly anterior). They also prefer occlusal surfaces and thereby appear to preserve a principle which had some justification in childhood but loses its validity in coming of age: Children learn that caries is the most important oral disease and that it manifests primarily at occlusal surfaces. This is true for children. In adulthood, however, caries manifests primarily at lateral and proximal surfaces [15–17] and the risk of developing gum disease and periodontitis increases and exceeds that of caries [2, 5]. It is important, that this change of principles is understood when entering adulthood. The current study indicates, however, that this is not the case. This assumption is supported by another representative study [25]. In that study, even middle-aged adults believed caries to be more important and prevalent than periodontal disease. Further, they indicated that it would be very important to clean one’s occlusal surfaces in order to prevent periodontitis [25]. More efforts should thus be undertaken to explain to adolescents (and adults) this change of principle.

The present investigation shows that many future efforts are necessary in order to better understand peoples’ oral hygiene behavior and how they could be taught the capability of achieving oral cleanliness. This is not impossible as demonstrated by a recent study showing that dental professionals already manage to keep their teeth clean and healthy [26]: Thus, it can be learned.

## Additional file

Additional file 1: Appendix: Flow diagram of participant recruitment. Delineates the recruitment of the two cohorts in detail: number of persons contacted, responding/not responding, excluded, included, examined, with no appointment. (DOCX 182 kb)

## Abbreviations

HB: Helen Blättermann (co-author)
ICC: Intraclass correlation
MPI: Marginal Plaque Index
QIT-S: Quality Index of toothbrushing regarding brushing time in sextants
RCT: Randomized controlled trial
SD: Standard deviation
SE: Stefanie Ebel (co-author)
