# Supplementary figures and images for: Toothbrushing: to the best of one’s abilities is possibly not good enough
Source: BMC Oral Health. 2018 Oct 19;18:167. doi: 10.1186/s12903-018-0633-0 (PMC6194646; doi:10.1186/s12903-018-0633-0)

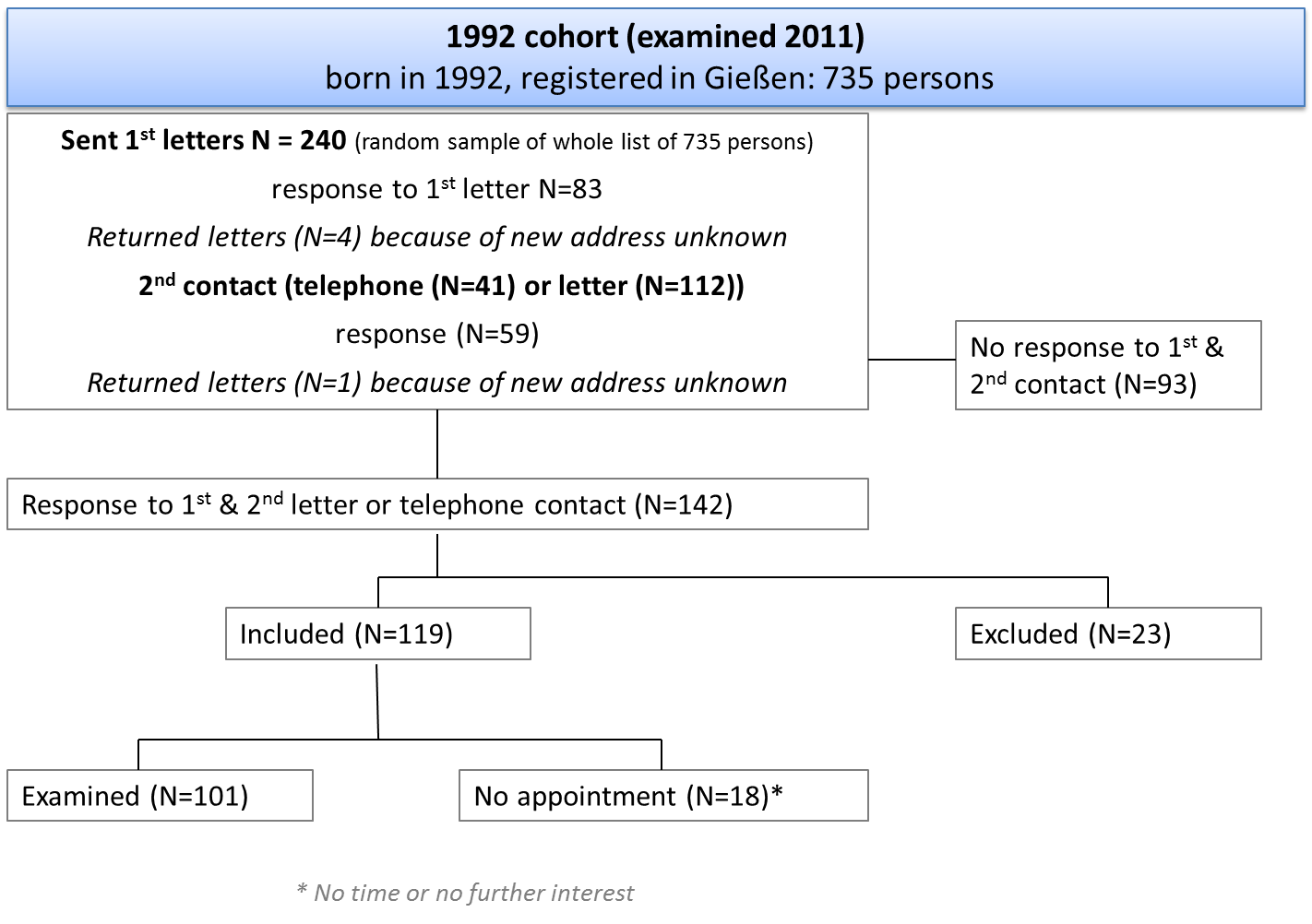


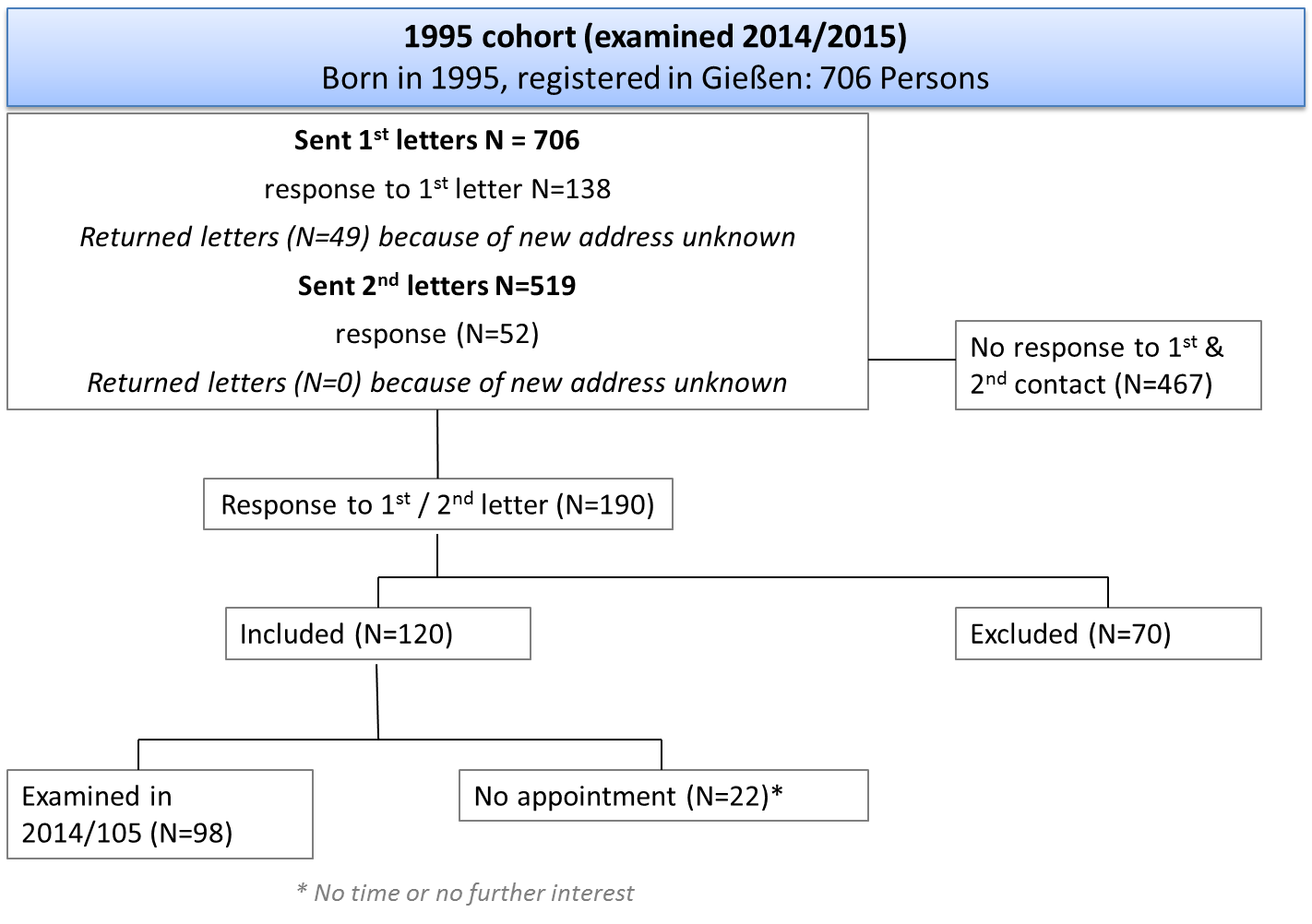

Supplement: Supplementary file 1 — Appendix: Flow diagram of participant recruitment. Delineates the recruitment of the two cohorts in detail: number of persons contacted, responding/not responding, excluded, included, examined, with no appointment. (DOCX 182 kb) [file 12903_2018_633_MOESM1_ESM.docx]
